# Targeted activation of AMPK by GSK621 ameliorates H_2_O_2_-induced damages in osteoblasts

**DOI:** 10.18632/oncotarget.14454

**Published:** 2017-01-02

**Authors:** Weidong Liu, Li Mao, Feng Ji, Fengli Chen, Yuedong Hao, Gang Liu

**Affiliations:** ^1^ Department of Orthopedics, Huai’an First People’s Hospital, Nanjing Medical University, Huai’an, China; ^2^ Department of Endocrinology, Huai’an First People’s Hospital, Nanjing Medical University, Huai’an, China; ^3^ Clinical Laboratory, Huai’an First People’s Hospital, Nanjing Medical University, Huai’an, China

**Keywords:** osteonecrosis, osteoblasts, AMPK, GSK621, oxidative stress

## Abstract

GSK621 is a novel AMP-activated protein kinase (AMPK) activator. This study tested its potential cytoprotective effect in hydrogen peroxide (H_2_O_2_)-treated osteoblasts. In cultured MC3T3-E1 osteoblastic cells and primary murine osteoblasts, GSK621 significantly attenuated H_2_O_2_-induced cell death and apoptosis. AMPK activation was required for GSK621-induced osteoblast cytoprotection. Inhibition of AMPK, by AMPKα1 T172A mutation or shRNA silence, almost completely blocked GSK621-induced osteoblast cytoprotection. Reversely, introduction of a constitutively-active AMPKα1 (T172D) alleviated H_2_O_2_ injuries in MC3T3-E1 cells. Further, GSK621 increased nicotinamide adenine dinucleotide phosphate (NADPH) content in osteoblasts to inhibit H_2_O_2_-induced reactive oxygen species (ROS) production. Meanwhile, GSK621 activated cytoprotective autophagy in the osteoblasts. On the other hand, pharmacological inhibition of autophagy alleviated GSK621-mediated osteoblast cytoprotection against H_2_O_2_. These results suggest that targeted activation of AMPK by GSK621 ameliorates H_2_O_2_-induced osteoblast cell injuries.

## INTRODUCTION

Osteoblasts are important for the bone formation and remodeling [1, 2]. Yet, these mesenchymal progenitor cells-derived cells are also the main target cells of oxidative stress [1, 2, 5]. Increased reactive oxygen species (ROS) production will lead to oxidative stress, causing osteoblast cell damage and apoptosis [6, 7]. Hydrogen peroxide (H_2_O_2_) is often added to cultured osteoblasts to establish a cellular model of osteonecrosis [8–11]. For many years, our group [12–15] has been focusing on indentifying novel molecular targets to promote osteoblast cell survival.

AMP-activated protein kinase (AMPK) is a master regulator of cellular metabolism and energy [16]. It plays a pivotal function in maintaining cell energy balance [16]. Existing studies have suggested that AMPK activation could also promote cell survival [17]. Recent literatures investigated the potential functions of AMPK in osteoblasts, and demonstrated that activating AMPK, either genetically or pharmacologically, could protect osteoblasts from oxidative stress and dexamethasone [12, 18–20]. Therefore, AMPK is a valuable pro-survival target at least in osteoblasts [12, 18–20].

Multiple AMPK activators of different mechanisms of actions have been developed thus far, many of them activate AMPK though increasing the AMP:ATP ratio, such as AICAR [21, 22]. Others, however, provoke AMPK activation by directly inducing AMPKα1 phosphorylation at Thr-172, *i.e*. Compound 13 [21–24]. Recent studies have developed GSK621 as a novel AMPK activator [25]. Its potential activity in osteoblasts has not been tested thus far. In this study, we show that GSK621 activates AMPK signaling and potentially inhibits H_2_O_2_-induced oxidative damages in cultured osteoblasts.

## RESULTS

### GSK621 protects osteoblasts from H_2_O_2_

**Figure 1:**
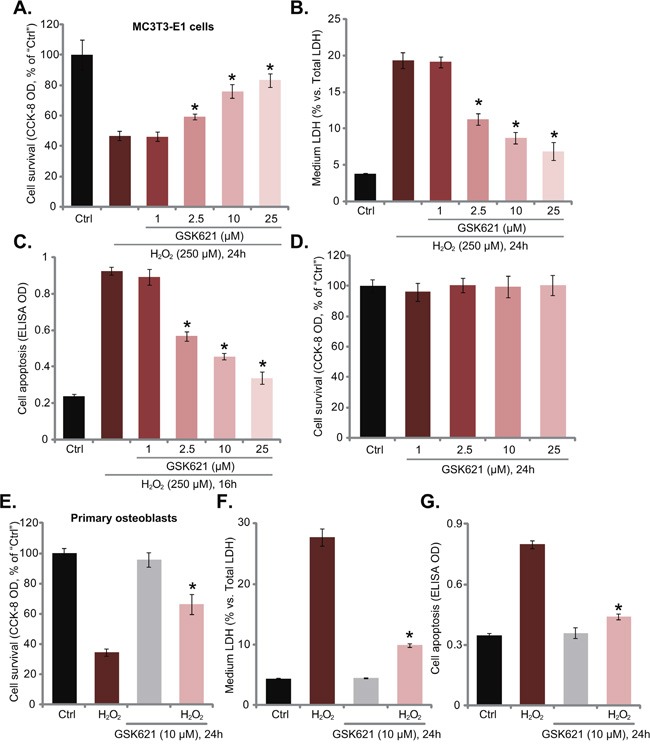
GSK621 protects osteoblasts from H_2_O_2_ MC3T3-E1 osteoblastic cells **A-D**. or the primary murine osteoblasts **E-G**. were treated with hydrogen peroxide (“H_2_O_2_”, 250 μM) with/out GSK621 at applied concentration, cells were then cultured for additional 16/24 hours; Cell survival (CCK-8 assay, A, D and E), cell death (LDH release assay, B and F) and apoptosis (Histone DNA ELISA assay, C and G) were tested. Data are shown as mean (n=5) ± standard deviation (SD). “Ctrl” stands for medium treatment control (Same for all figures). Experiments in this figure were repeated for three times, and similar results were obtained. *p<0.05 *vs*. H_2_O_2_ only group.

The current study aims to understand the potential effect of GSK621 on oxidative-stressed osteoblasts. CCK-8 viability results in Figure 1A demonstrated that H_2_O_2_ (250 μM, 24 hours) treatment in MC3T3-E1 osteoblastic cells [15] induced over 50% cell viability reduction. Significantly, co-treatment with GSK621 at 2.5-25 μM dramatically attenuated H_2_O_2_-induced MC3T3-E1 cell viability reduction (Figure 1A). LDH release results in Figure 1B confirmed H_2_O_2_ (250 μM)-induced MC3T3-E1 cell death, which was again largely attenuated with co-treatment of GSK621 (2.5-25 μM). Meanwhile, H_2_O_2_ (250 μM)-induced MC3T3-E1 cell apoptosis, tested by Histone DNA ELISA assay [12, 13], was also significantly alleviated by GSK621 co-treatment (Figure 1C). The anti-H_2_O_2_ activity of GSK621 in MC3T3-E1 cells was dose-dependent (Figure 1A-1C). At a low concentration (1 μM), GSK621 was invalid to inhibit H_2_O_2_ damages (Figure 1A-1C). Notably, treatment with GSK621 alone at tested concentrations failed to induce survival change (Figure 1D) and apoptosis (Data not shown) in MC3T3-E1 cells.

Using the methods described [12–15], we also established primary murine osteoblasts. H_2_O_2_ (250 μM) treatment in these primary cells also induced viability reduction (Figure 1E), cell death (Figure 1F) and apoptosis (Figure 1G). Remarkably, GSK621 (10 μM) co-administration significantly alleviated H_2_O_2_-induced damages of the primary osteoblasts (Figure 1E-1G). GSK621 (10 μM) alone again didn’t affect survival and apoptosis of the primary cells (Figure 1E-1G). These results show that GSK621 indeed protects osteoblasts from H_2_O_2_.

### GSK621-mediated osteoblast cytoprotection requires AMPK activation

**Figure 2:**
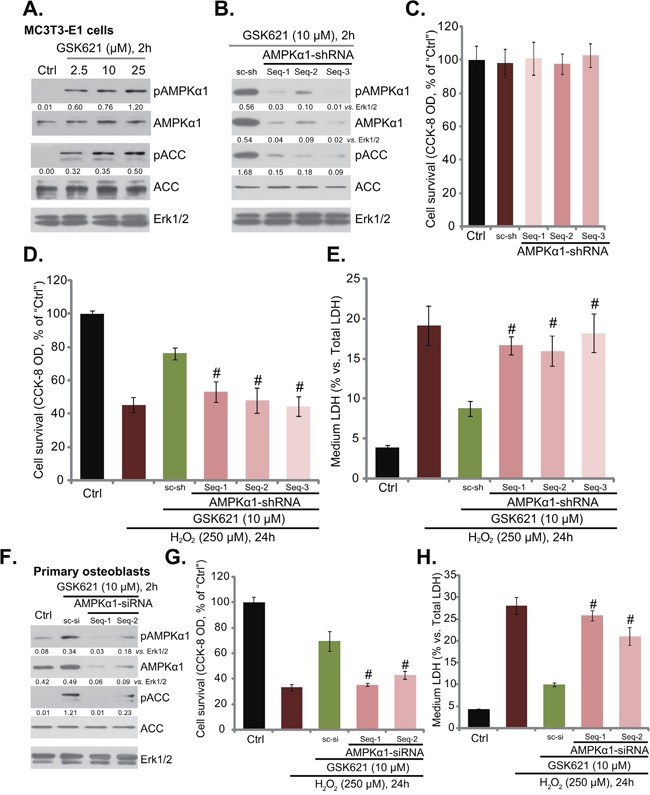
GSK621-mediated osteoblast cytoprotection requires AMPK activation MC3T3-E1 cells were treated with GSK621 at applied concentrations for 2 hours, expression of listed proteins was shown **A**. MC3T3-E1 cells, expressing listed lentiviral AMPKα1 shRNA (“Seq-1/-2/3”) or scramble control shRNA (“sc-sh”), were subjected to Western blotting assay of listed proteins **B**.; These cells were also treated with/out H_2_O_2_ (250 μM) or plus GSK621 (10 μM) for 24 hours; Cell viability (CCK-8 assay, **C**. and **D**.) and cell death (LDH release assay, **E**. were tested. Primary murine osteoblasts, transfected with indicated AMPKα1 siRNA (“Seq-1/-2”) or scramble control siRNA (“sc-si”), were treated with GSK621 (10 μM) for 2 hours, expression of listed proteins was tested **F**. Cells were also stimulated with H_2_O_2_ (250 μM) for 24 hours, and cell survival **G**. and cell death **H**. were tested. AMPKα1 (“p-” and total) or p-ACC were quantified. Data are shown as mean (n=6) ± SD. Experiments in this figure were repeated for three times, and similar results were obtained. ^#^*p*<0.05 vs. “sc-sh”/“sc-si” group.

**Figure 3:**
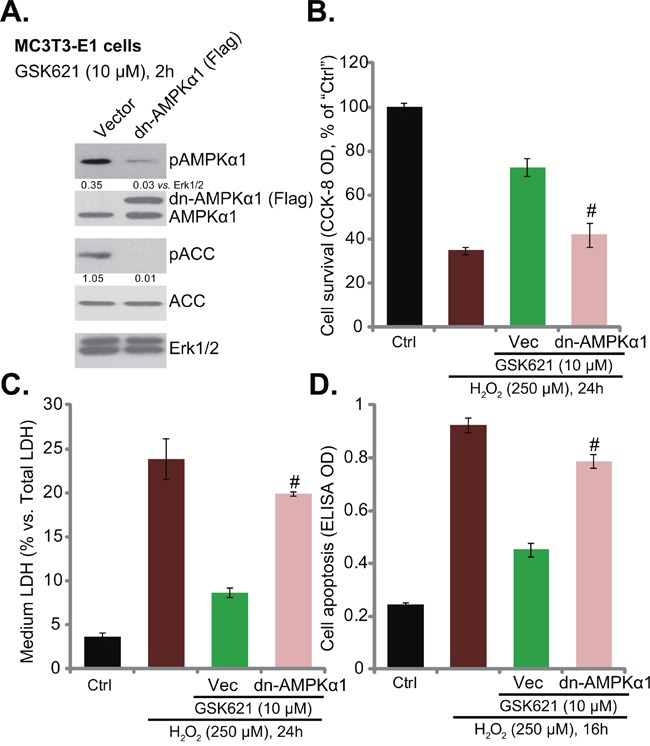
AMPKα1 dominant negative mutation abolishes GSK621-induced osteoblast cytoprotection Neomycin-selected stable MC3T3-E1 cells expressing dominant negative AMPKα1 (T172A) construct (“dn-AMPKα1”, Flag-tagged) or the empty vector (pSuper-Flag, “Vec”) were treated with GSK621 (10 μM) for 2 hours, expression of listed proteins was tested **A**. Cells were also subjected to H_2_O_2_ (250 μM) stimulation; Cell survival (CCK-8 assay, **B**. Cell death (LDH release assay, **C**. and apoptosis (Histone-DNA ELISA assay, **D**. were tested. Data are shown as mean (n=4) ± SD. p-AMPKα1 and p-ACC were quantified. Experiments in this figure were repeated for three times, and similar results were obtained. ^#^*p*<0.05 vs. “Vec” group.

GSK621 is a newly-developed AMPK activator [25–27], we therefore tested AMPK signaling in GSK621-treated cells. As shown in Figure 3A, treatment with GSK621 (at 2.5-25 μM, 2 hours) in MC3T3-E1 cells induced significant AMPK activation, which was tested by phosphorylation (“p”) of AMPKα1 (Thr-172) and its major downstream target protein ACC (acetyl-CoA carboxylase, Ser-79) [12]. Expression of total AMPKα1 and ACC was not changed following the GSK621 treatment (Figure 3A). To study the link between GSK621-induced AMPK activation and osteoblast cytoprotection, shRNA strategy [12] was applied to silence AMPK signaling. In the study, a total of three different lentiviral AMPKα1 shRNAs (“Seq-1/-2/-3”) were designed (See Methods), and each of them potently downregulated AMPKα1 in MC3T3-E1 cells (Figure 2B). Consequently, GSK621-induced AMPK activation, or p-AMPKα1/p-ACC, was almost abolished in AMPKα1-silenced cells (Figure 2B). Importantly, although the AMPKα1 shRNAs alone didn’t affect MC3T3-E1 cell survival (Figure 2C), they almost abolished GSK621-mediated osteoblast cytoprotection against H_2_O_2_ (250 μM) (Figure 2D and 2E). In another words, GSK621 was pretty much invalid against H_2_O_2_ when AMPKα1 was silenced (Figure 2D and 2E).

In the primary murine osteoblasts, two non-overlapping AMPKα1 siRNAs (“Seq-1/-2”) were utilized to knockdown AMPKα1. As demonstrated, the two applied siRNAs knocked down AMPKα1 in the primary cells (Figure 2F). GSK621 (10 μM, 2 hours)-induced AMPK activation, or p-AMPKα1/p-ACC, was also dramatically inhibited with AMPKα1 siRNA knockdown (Figure 2F). Similarly, GSK621 was also largely ineffective when AMPKα1 was silenced (Figure 2G and 2H). Seq-1 AMPKα1 siRNA was slightly more efficient than Seq-2 siRNA in downregulating AMPKα1 (Figure 2F). It was also more efficient in shutting down GSK621-mediated cytoprotection (Figure 2G and 2H). Together, these results indicate that AMPK activation is required for GSK621-induced osteoblast cytoprotection against H_2_O_2_.

### AMPKα1 dominant negative mutation abolishes GSK621-induced osteoblast cytoprotection

To further confirm the requirement of AMPK activation in GSK621-induced actions in osteoblasts, a dominant negative AMPKα1 (T172A) construct (“dn-AMPKα1”, Flag-tagged) [12] was utilized. The mutant AMPKα1 was introduced to MC3T3-E1 cells. Via neomycin selection, stable MC3T3-E1 cells with the mutant AMPKα1 were established. Western blotting assay results in Figure 3A confirmed dn-AMPKα1 expression in the stable cells. Further, GSK621 (10 μM, 2 hours)-induced AMPK activation (p-AMPKα1/p-ACC) was almost abolished in dn-AMPKα1-expressing cells (Figure 3A). More importantly, GSK621-induced osteoblast protection against H_2_O_2_ was also largely inhibited with AMPKα1 mutation (Figure 3B-3D). GSK621-induced pro-survival (Figure 3B), anti-death (Figure 3C) and anti-apoptosis (Figure 3D) actions in H_2_O_2_-treated cells were significantly attenuated. These results again confirm that AMPK activation is required for GSK621-mediated osteoblast cytoprotection against H_2_O_2_.

### Forced-activation of AMPK protects osteoblasts from H_2_O_2_, taking over GSK621’s actions

**Figure 4:**
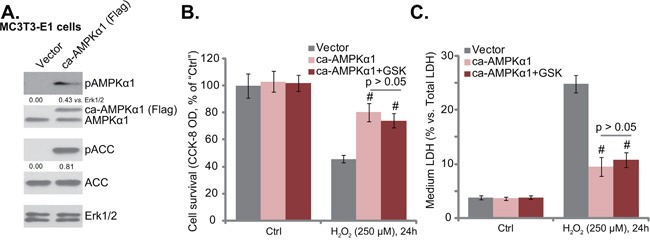
Forced-activation of AMPK protects osteoblasts from H_2_O_2_, taking over GSK621’s actions Puromycin-selected stable MC3T3-E1 cells, expressing the constitutively-active AMPKα1 (T172D) construct (“ca-AMPKα1”, Flag-tagged) or the empty vector (pSuper-Flag, “Vector”), were subjected to Western blotting assay of listed proteins **A**. Above cells were also treated with H_2_O_2_ (250 μM) or plus GSK621 (10 μM) for 24 hours; Cell survival (CCK-8 assay, **B**. and cell death (LDH release assay, **C**. were tested. Data are shown as mean (n=4) ± SD. p-AMPKα1 and p-ACC were quantified. Experiments in this figure were repeated for four times, and similar results were obtained. ^#^*p*<0.05 vs. “Vector” group.

Next, a constitutively-active AMPKα1 (T172D) construct (“ca-AMPKα1”, Flag-tagged) [12] was introduced to MC3T3-E1 cells, and stable cell line with the ca-AMPKα1 was selected by puromycin. Western blotting results in Figure 4A confirmed expression of ca-AMPKα1 in the stable cells. As expected, AMPK was constitutively active in these cells, and p-AMPKα1 and p-ACC level was high (Figure 4A). Compared to the vector control MC3T3-E1 cells, the ca-AMPKα1-expressing cells were protected from H_2_O_2_, presenting with significantly less viability reduction (Figure 4B) and cell death (Figure 4C) after H_2_O_2_ treatment. Significantly, in ca-AMPKα1-expressing MC3T3-E1 cells, GSK621 (10 μM) could not further protect cells from H_2_O_2_ (Figure 4B and 4C). Thus, ca-AMPKα1 expression took over GSK621’s actions and inhibited H_2_O_2_ damages in MC3T3-E1 cells. These results against indicate that activation of AMPK is required for GSK621-mediated osteoblast cytoprotection.

### GSK621 increases NAPDH content and inhibits H_2_O_2_-induced oxidative stress

**Figure 5:**
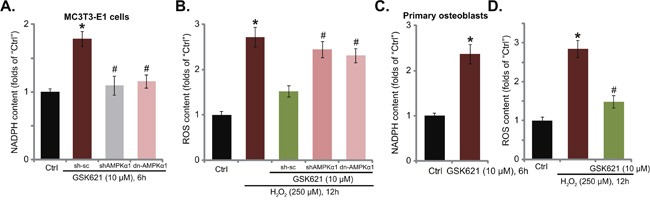
GSK621 increases NAPDH content and inhibits H_2_O_2_-induced oxidative stress MC3T3-E1 cells, expressing scramble control shRNA (“sc-sh”), AMPKα1 shRNA (Seq-1, “sh AMPKα1”) or dominant negative AMPKα1 (T172A) (“dn-AMPKα1”), were treated with GSK621 (10 μM) or plus H_2_O_2_ (250 μM) for applied time; Relative NAPDH level **A**. and ROS content **B**. were tested by the listed assays. The primary murine osteoblasts were treated with GSK621 (10 μM) or plus H_2_O_2_ (250 μM) for applied time, relative NAPDH level **C**. and ROS content **D**. were tested. Data are shown as mean (n=3) ± SD. Experiments in this figure were repeated for four times, and similar results were obtained. *p<0.05 vs. “Ctrl” group. ^#^*p*<0.05 vs. “H_2_O_2_” only group.

Recent studies have proposed an anti-oxidant function of AMPK under many stress conditions. Activated AMPK may increase nicotinamide adenine dinucleotide phosphate (NADPH) content to inhibit ROS production and accumulation [19, 20, 28, 29]. Here, GSK621 treatment also increased NADPH level in MC3T3-E1 cells (Figure 5A), which was blocked by AMPKα1 shRNA knockdown or dominant negative mutation (Figure 5A). Intriguingly, H_2_O_2_-induced ROS production was also largely inhibited with co-treatment of GSK621 (Figure 5B). More importantly, the anti-oxidant activity by GSK621 was nullified with AMPKα1 shRNA or dominant negative mutation (Figure 5B). Therefore, GSK621 inhibits H_2_O_2_-induced ROS production in an AMPK-dependent manner. In the primary murine osteoblasts, GSK621 (10 μM) similarly increased NAPDH content (Figure 5C) and inhibited H_2_O_2_-induced ROS production (Figure 5C). These results show that GSK621 increases NAPDH content and inhibits H_2_O_2_-induced oxidative stress in osteoblasts.

### GSK621 activates cytoprotective autophagy in osteoblasts

**Figure 6:**
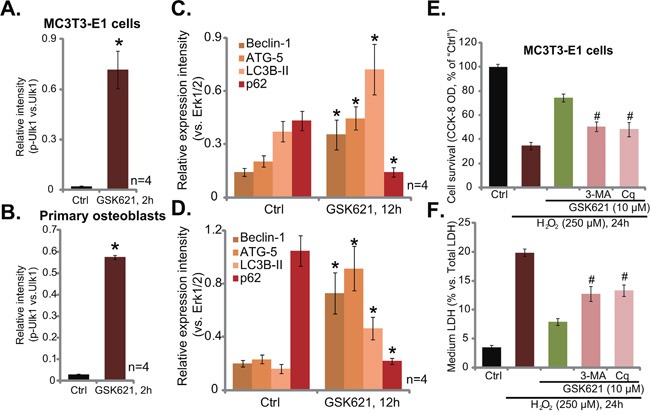
GSK621 activates cytoprotective autophagy in osteoblasts MC3T3-E1 cells (A and C) or the primary murine osteoblasts (B. and D.) were treated with GSK621 (10 μM) for designated time; Expression of listed proteins was tested by Western blotting assay, results were quantified (**A-D**). MC3T3-E1 cells, pre-treated for 1 hour with 3-methyladenine (3-MA, 0.5 mM) or chloroquine (Cq, 10 μM), were then treated with H_2_O_2_ (250 μM) or plus GSK621 (10 μM) for 24 hours; Cell viability (CCK-8 assay, **E**) and cell death (LDH release assay, **F**) were tested. Data are shown as mean (n=4) ± SD. Experiments in this figure were repeated for four times, and similar results were obtained. ^#^*p*<0.05 vs. “GSK621” only group.

AMPK could also provoke autophagy to promote cell survival [19, 30, 31]. AMPK is shown to directly phosphorylate and activate its key downstream Ulk1 to initiate autophagy, which is cytoprotective [19, 30, 31]. Here, in both MC3T3-E1 cells (Figure 6A) and primary murine osteoblasts (Figure 6B), GSK621 (10 μM, 2 hours) induced significant Ulk1 phosphorylation at Ser-317 (Results were quantified), the site that can only be activated by AMPK [30]. Subsequently, expression of autophagy-associated proteins, including Beclin-1, autophagy-related homologue 5 (ATG-5) and light chain 3B-II (LC3B-II), was significantly increased, while p62 was degradated (See quantified results in Figure 6C-6D). These results suggested significant autophagy activation in GSK621-treated cells [32–34]. Remarkably, as shown in Figure 6E and 6F, GSK621 (10 μM)-induced osteoblast cytoprotection against H_2_O_2_ was compromised in the presence of autophagy inhibitor 3-methyaldenine (3-MA) and chloroquine (Cq) [32, 35]. Therefore, GSK621’s actions in MC3T3-E1 cells was weakened when autophagy was pharmacologically inhibited (Figure 6E and 6F). These results indicate that GSK621 activates cytoprotective autophagy in osteoblasts.

## DISCUSSION

The results of the current study suggest that AMPK activation is required for GSK621-induced osteoblast cytoprotection against H_2_O_2_. shRNA stable knockdown of AMPKα1 almost completely blocked GSK621-induced AMPK activation and osteoblast cytoprotection. Similarly, AMPK in-activation via intruding the dominant negative mutant AMPKα1 (T172A) also nullified GSK621-mediated anti-H_2_O_2_ actions in osteoblasts. Reversely, forced-activation of AMPK via introducing the ca-AMPKα1 mimicked GSK621’s actions and alleviated H_2_O_2_-induced osteoblast cell death. Remarkably, GSK621 was unable to further protect osteoblasts with ca-AMPKα1 expression. All these results imply that activation of AMPK is indispensable for GSK621-induced cytoprotection in osteoblasts.

Several mechanisms are responsible for AMPK-mediated cytoprotection. For example, AMPK could activate cytoprotective autophagy, which recycles cellular components to provide nutrition for cell survive [36, 37]. AMPK is shown to directly phosphorylate Ulk1 to initiate cell autophagy [36, 37]. Further, AMPK could also function as an anti-oxidant protein, via increasing NADPH content and limiting ATP consumption [28]. In addition, under starvation condition, AMPK activation could also protect cells through in-activating mTOR complex 1 (mTORC1) [38].

Here, we found that GSK621 induced NADPH production to inhibit H_2_O_2_-induced ROS production in osteoblasts. Such effects by GSK621 were dependent on AMPK activation. As AMPKα1 dominant negative mutation (T172A) or shRNA stable knockdown almost abolished GSK621-induced NADPH production and ROS scavenging activity. Our results here are consistent with recent findings showing ROS clearance by several different AMPK activators in osteoblasts [12, 18, 19]. Meanwhile, we showed that GSK621 treatment in the osteoblasts also activated cytoprotective autophagy, which was evidenced by Ulk1 phosphorylation at Ser-317, upregulation of Beclin-1, ATG-5 and LC3B-II, as well as degradation of p62. Importantly, autophagy inhibitors alleviated GSK621-mediated osteoblast cytoprotection. Together, ROS clearance and autophagy activation could be two major downstream targets of AMPK in mediating GSK621’s cytoprotection in osteoblasts. Although, the detailed underlying mechanisms may warrant further investigations.

## MATERIALS AND METHODS

### Chemicals, regents and antibodies

GSK621 was purchased from Gao-Chem (Shanghai, China). H_2_O_2_, puromycin, 3-methyaldenine (3-MA) and chloroquine (Cq) were purchased from Sigma Chemicals (St. Louis, MO). Anti-AMPKα1, acetyl-CoA carboxylase (ACC) and Beclin-1, autophagy-related homologue 5 (ATG-5), light chain 3B-II (LC3B-II), p62 and Erk1/2 antibodies were purchased from Santa Cruz Biotech (Santa Cruz, CA). Antibodies against phospho(p)-AMPKα1 (Thr 172), p-ACC (Ser 79), p-S6K1 (Thr-389), p-S6 (Ser-235/236) and total S6K1 were obtained from Cell Signaling Tech (Denver MA).

### Culture of MC3T3-E1 osteoblastic cells

The murine calvaria-derived osteoblastic-like MC3T3-E1 cells were cultured and differentiated as described in our previous studies [13, 14].

### Isolation and primary culture of murine osteoblasts

The isolation and primary culture of murine osteoblasts were described previously [13, 14]. The animal protocols were approved by Institutional Animal Care and Use Committee (IACUC) and Institutional Ethics Committee.

### Cell survival assay

Cell Counting Kit-8 (CCK-8, Dojindo Laboratories, Kumamoto, Japan) was applied to test survival of osteoblasts with applied treatment/s. The detailed protocol was described in our previous studies [13, 14].

### Cell apoptosis assay

To test cell apoptosis, the histone-DNA ELISA plus kit (Roche, Palo Alto, CA) [13, 14] was applied. ELISA OD at 450 nm was recorded as the indicator of cell apoptosis [13, 14].

### Cell death assay

Analyzing cell death by measuring lactate dehydrogenase (LDH) content in the conditional medium was through a two-step enzymatic reaction LDH assay kit (Takara, Tokyo, Japan), as described [13, 14].

### Western blotting assay

As described previously [13, 14], 30 μg protein lysates per sample were separated by SDS-PAGE gel, and were transferred to the PVDF membranes. These blots were then blocked, and were subsequently incubated with primary and specific secondary antibodies. Protein bands were visualized via ECL reagents (Pierce, Shanghai, China). Band intensity of total gray was quantified via the ImageJ software [13, 14].

### AMPKα1 stable knockdown

A set of three different murine AMPKα1 shRNAs with non-overlapping sequences (“Seq-1/-2/-3”, Genepharm, Shanghai, China) were constructed into the GV428 lentiviral vector (Genepharm), containing a puromycin-resistance gene and the Flag-tag. MC3T3-E1 cells were cultured with 50% confluence. The lentiviral shRNA (5 μL/mL) was added directly to the cultured cells for 24 hours. Cells were then subjected to puromycin (0.5 μg/ml) selection for the other 24 hours. Afterwards, AMPKα1 expression was detected by Western blotting assay. Control cells were infected with scramble control shRNA (Genepharm).

### AMPKα1 mutation

As described previously [12], the dominant negative AMPK-α1 (dn-AMPK-α1, T172A) construct or the constitutively-active mutant AMPKα1 (T172D, ca-AMPKα1) was transfected to the MC3T3-E1 cells (0.20 μg/mL \ each) via the Lipofectamine 2000 reagent [39]. Stable cells were selected via neomycin (1 μg/mL, for dn-AMPK-α1) or puromycin (0.5 μg/mL, for ca-AMPKα1) for a total of two weeks. AMPKα1 expression and phosphorylation in the stable cells were detected by Western blotting assay.

### AMPKα1 siRNA knockdown

The two non-overlapping murine AMPKα1 siRNAs (“Seq-1/Seq-2”) and the scramble control siRNA were synthesized by Genepharm. Transient transfection (100 nM, 24 hours) was performed by the Lipofectamine 2000 reagents according to the manufacturer’s instructions. Transfection efficiency was determined by Western blotting assay.

### Assay of intracellular ROS level

The cellular reactive oxygen species (ROS) content was detected via the 2′, 7′-dichlorofluorescein diacetate (H2-DCFDA; Abcam, Shanghai, China) FACS method as described [18]. ROS level in treatment group was normalized to that of control group.

### NADPH content assay

The intracellular NADPH content was tested via the previously described enzymatic cycling method [18, 19, 28]. Briefly, after treatment, twp million cells per sample were lysed, and the supernatant was incubated at 60°C for 30 min before NADP-cycling buffer plus glucose-6-phosphate dehydrogenase (G6PD, Sigma) [19] were added. Afterwards, glucose 6-phosphate (G6P, Sigma) was added to the mixture, and the change in absorbance at 570 nm was measured at 30°C. NADPH level in treatment group was normalized to that of control group.

### Statistical analysis

Comparisons between groups were performed via one-way ANOVA and the Newman-Keuls test (SPSS 18.0). p values < 0.05 were considered statistically significant.

## CONCLUSIONS

Together, we conclude that targeted activation of AMPK by GSK621 significantly alleviates H_2_O_2_-induced damages in osteoblasts. GSK621 and other AMPK activators might have translational value for treatment oxidative stress-associated osteonecrosis.
